# Efficient detection of QTL with large effects in a simulated pig-type pedigree using selective genotyping

**DOI:** 10.1186/1753-6561-3-s1-s8

**Published:** 2009-02-23

**Authors:** Henri CM Heuven, John WM Bastiaansen, Stéphanie M van den Berg

**Affiliations:** 1 Clinical Sciences of Companion Animals, Faculty of Veterinary Medicine, Utrecht University. P.O. box 80163, 3508 TD Utrecht, The Netherlands; 2 Animal Breeding and Genomics Centre, Wageningen University, P.O.-box 338, 6700AH Wageningen, the Netherlands

## Abstract

**Background:**

The ultimate goal of QTL studies is to find causative mutations, which requires additional expression studies. Given the limited amount of time and funds, the smart option is to identify the most important QTL with minimal effort. A cost-effective solution is to genotype only those animals with high or low phenotypic values or DNA-pools of these individuals. A two-stage genotyping strategy was applied on samples in the tails of the distribution of breeding values.

**Results:**

The tail-analysis approach identified eight out of the 19 QTL in the first stage, explaining about half of 98% of the genetic variance. Four additional QTL with small effects were found in the second stage.

**Conclusion:**

The two-stage genotyping strategy with selective genotyping detected regions with highly significant QTL useful for further fine-mapping. The large reduction in costs allows for follow-up expression and functional studies.

## Background

Discovery and subsequent validation of causative mutations affecting complex traits require identification and fine-mapping of QTL followed by expression and functional studies. Given the limited amount of time and funds, the challenge is to identify the most important QTL with minimal effort.

A cost-effective strategy is to reduce genotyping costs by only genotyping individuals with high and low phenotypic values, or to genotype pools of these individuals. Tail analysis, bulked segregant analysis and selective DNA pooling have been advocated by Hillel et al. [1], Michelmore et al. [2] and Darvasi and Soller [3]. More recently Korol et al. [4] improved on the latter method by studying fractioned DNA pooling. Disadvantages to genotyping tails or pools are the number of traits that can be studied with the selected genotypes, separate high/low tails or pools have to be made for each trait, and non-optimal use of haplotype information. Wang et al. [5] improved on statistical methods developed by Dekkers [6] for interpretation of results obtained by DNA pooling.

Commercial breeding pedigrees present a situation where phenotypes are abundant, across many generations. In such a situation, selective genotyping is an important step in setting up a cost effective QTL study. This study implements a two-stage strategy. First, genotypes on a large SNP panel are obtained for highly informative individuals, that is, individuals with extreme breeding values. High and low phenotype animals are selected within each sire-dam pairing in order to control for stratification.

The objective is to identify major segregating QTL in a simulated pig-type pedigree with minimal effort both in terms of genotyping and analysis.

## Methods

In a four generation pedigree, 45 sires produced 100 offspring each. Each sire was mated to 10 dams with 10 progeny each. Sires and dams of the base generation were unknown. All 4665 animals were phenotyped for a quantitative trait (TRT). Six thousand equally distributed (0.1 cM) SNPs were available for genotyping, located on 6 chromosomes of 100 cM each. A full description of the dataset can be found at the website of XII^th ^QTLmas workshop [7].

### Genotyping strategy

#### Stage 1

For each sire, the offspring with the highest and the lowest EBV within a set of full sibs (i.e. per dam) were included into the high tail (H-tail) and the low tail (L-tail) respectively. Since there were 10 dams per sire there were 10 animals in either tail for each sire. Only sires with progeny that have phenotype records were used.

For each SNP and for each sire, the frequencies of the '1' and '2'-alleles in the high and low tail were determined and submitted to a χ^2 ^(1) test. SNPs with a Pearson statistic exceeding 10 (nominal p-value < 0.0016) were considered putative. A Pearson χ^2 ^value exceeding 10 required that the counts of the allele in either tail differed by at least 10. A difference of 10 alleles suggested linkage between a QTL and this SNP in the sire, assuming equal contributions of the dam's alleles to both tails. A Chi-square test was appropriate under the null hypothesis of no association and the assumption that both sires and dams were sampled randomly from the population with respect to their SNP genotypes.

#### Stage 2

When multiple segregating SNPs occur in a small region then this region was considered likely to contain a QTL. Genotypes of all putative SNPs were subsequently obtained for all animals with phenotype records and an association was determined by applying the following model:

(1) TRT = μ + Zu + SNP + e

Where:

TRT = trait value

μ = overall mean

Z = incidence matrix linking polygenic effects to individuals

u = polygenic effect ~*N*(0, Aσ_a_^2^) with A as the additive genetic relationship matrix

SNP = effect of single SNP (four classes: 11, 12, 21, 22)

e = residual effects ~*N*(0, Iσ_e_^2^); with I as the Identity matrix

Model selection, i.e. which SNP(s) needs be included in the model, was determined by region. Forward stepwise regression was applied to identify markers with a large effect.

For fine-mapping LDLA-software was used [8]. was applied to identify markers with a significant effect. This was done per region, where the regions were those identified in stage 1. In LDLA a QTL was fitted at the midpoint of each bracket formed by each pair of adjacent SNPs. Phased adjacent markers defined a haplotype. The genotypic data was already phased but with 100 progeny per sire phasing should be straightforward. LDLA utilizes the same model as described above except that SNP was now a random haplotype effect instead of a fixed individual SNP effect. Both linkage and segregation information from sires and dams contributed to indicated the best location per region by using the covariance among founder haplotypes to account for linkage information and covariance among parent and offspring haplotypes to account for segregation information [9]. At each bracket midpoint the likelihood of the model was compared to a model with a polygenic effect only to determine the significance. Threshold values were corrected for multiple testing [10].

## Results

### Stage 1

114 putative markers significantly (p < 0.0016) differed in frequency between the high and low tail in at least one sire family (Table 1). Five markers were significantly different between tails in 2 sire families, but all other putative markers were discovered from the difference between pools in only a single sire family. The putative markers were identified in tails from 21 sires of which 8 sires segregated only for one putative marker. In 24 sire families no SNPs were identified as putative. Most of the 114 putative markers occurred in groups of positions, indicating regions where QTL might be segregating.

**Table 1:** Putative markers identified using Chi-square tests on high and low tails for each sire.

Marker	Sire ID	# 2's low-pool	# 2's high-pool	Chi-square	Marker	Sire ID	# 2's low-pool	# 2's high-pool	Chi-square
232	8	17	7	10.4	2277	1034	16	6	10.1
274	9	4	14	10.1	2692	2627	0	9	11.6
285	9	16	6	10.1	2733	12	7	17	10.4
289	9	4	14	10.1	3007	389	3	14	12.4
290	1117	17	7	10.4	3008	389	3	13	10.4
296	1117	7	17	10.4	3011	389	17	6	12.4
302	1117	3	13	10.4	3014	2483	3	13	10.4
361	15	14	4	10.1	3024	1493	17	7	10.4
386	1117	14	4	10.1	3029*	1493	13	3	10.4
396	15	16	5	12.1	3030	15	18	8	11.0
397	8	5	16	12.1	3031	2483	1	10	10.2
399	15	16	5	12.1	3032	2483	1	12	13.8
403	15	4	16	14.4	3033*	15	6	16	10.1
404	8	16	6	10.1	3034	2483	19	10	10.2
411	1117	16	6	10.1	3039	9	18	8	11.0
415	1117	16	6	10.1	3040	1493	6	16	10.1
416	1117	16	6	10.1	3043	1493	14	4	10.1
426	1662	10	19	10.2	3045	9	16	6	10.1
428	1662	10	1	10.2	3046	1493	6	16	10.1
437	1117	16	6	10.1	3047	2483	4	14	10.1
439	1117	16	6	10.1	3048	1493	6	16	10.1
442	1117	17	6	12.4	3049	2483	2	12	11.0
444	1117	16	6	10.1	3051	2483	1	10	10.2
450	1117	3	14	12.4	3056	9	16	6	10.1
452	1117	3	14	12.4	3058	9	16	6	10.1
455	1117	19	8	13.8	3061	389	4	14	10.1
457	1117	14	4	10.1	3062*	389	16	6	10.1
464	1117	19	10	10.2	3068*	389	4	14	10.1
466	1117	2	12	11.0	3079	2483	4	14	10.1
513	1117	0	9	11.6	3080	2483	2	12	11.0
534	1117	16	6	10.1	3082	9	16	6	10.1
726	222	10	19	10.2	3091	389	4	14	10.1
1125	1475	17	7	10.4	3151	222	18	7	12.9
1212	8	14	4	10.1	3159	222	16	6	10.1
1326	8	4	14	10.1	3177	389	14	3	12.4
1483	2008	14	4	10.1	3180	389	6	16	10.1
1498	2008	7	17	10.4	3182	1149	18	8	11.0
2160	1034	14	4	10.1	3192	389	6	17	12.4
2162	1034	6	17	12.4	3229	2	4	14	10.1
2166	1034	6	17	12.4	3479	2483	14	4	10.1
2173*	1	4	14	10.1	3487	2483	16	6	10.1
2176	1034	18	7	12.9	3506	529	17	7	10.4
2178	1	17	7	10.4	3514	389	18	7	12.9
2180	1034	1	11	11.9	3546	529	4	14	10.1
2182	1	3	13	10.4	3550	529	4	14	10.1
2183	1	2	13	12.9	3558	529	19	10	10.2
2185	1	14	4	10.1	3646	389	14	4	10.1
2187	1	4	14	10.1	3701	529	5	16	12.1
2189	1	2	13	12.9	3710	529	8	18	11.0
2190	1034	17	7	10.4	3714	529	7	18	12.9
2192	1	18	7	12.9	3716	529	12	2	11.0
2193	1034	18	6	15.0	3765	529	5	16	12.1
2196	1	14	4	10.1	3766	529	5	16	12.1
2219	1	2	13	12.9	3965	389	13	3	10.4
2220	1	18	8	11.0	4891	7	19	10	10.2
2221	1	18	8	11.0	5156	3127	14	4	10.1
2225	1	16	6	10.1	5330	2528	3	13	^10.4^

* Markers significantly segregating for 2 sires

### Stage 2

The next step was to obtain genotypes for all phenotyped animals for the putative markers identified in stage 1, in order to distinguish between truly associated markers and false positives. Individual marker association with the trait was calculated using model 1 (i.e. a model including each marker in turn as well as a polygenic effect). Table 2 summarizes these results. Table 3 shows the results of forward stepwise regression. In each subsequent analysis four SNPs with the most significant associations (F-statistics obtained after correcting for the previous entered SNPs) were added. The polygenic variance decreased indicating that 12 markers accounted for close to 30% of the genetic variance. The results of the third round indicate that on each of chromosomes 1, 2 and 4 there were regions with QTL. The size of the QTL can be deduced from the effects of the genotypes in round three (Table 4). With 100 progeny per sire, haplotypes could easily be determined and genotypes 12 and 21 could be distinguished in most cases. QTL with the largest effects are expected near SNP 415, 3033 and 3765. Except for SNP 513, heterozygous genotype effects were intermediate to the effects of the homozygous genotypes indicating that the QTL were additive.

**Table 2:** Significance of individual markers with all animals genotyped, corrected for polygenic effects.

SNP	F-statistic	σ ^2^_e_	σ^2^_a_	SNP	F-statistic	σ ^2^_e_	σ^2^_a_
232	3.59	3.13	1.34	2277	1.64	3.11	1.38
274	2.21	3.13	1.34	2692	2.57	3.12	1.36
285	3.93	3.11	1.37	2733	1.14	3.13	1.36
289	4.40	3.14	1.32	3007	0.96	3.13	1.36
290	2.83	3.11	1.38	3008	1.52	3.13	1.35
296	4.72	3.12	1.36	3011	0.85	3.13	1.36
302	2.95	3.10	1.39	3014	2.86	3.12	1.36
361	2.14	3.13	1.35	3024	6.30	3.12	1.35
386	2.39	3.12	1.35	3029	3.25	3.12	1.36
396	4.65	3.11	1.37	3030	22.97	3.10	1.31
397	10.33	3.13	1.31	3031	9.57	3.10	1.37
399	3.52	3.11	1.37	3032	15.25	3.10	1.34
403	3.79	3.11	1.38	3033	42.24	3.10	1.22
404	11.66	3.14	1.29	3034	0.49	3.12	1.38
411	2.56	3.11	1.38	3039	4.36	3.12	1.35
415	16.50	3.12	1.31	3040	4.60	3.12	1.35
416	8.79	3.13	1.31	3043	3.82	3.12	1.35
426	2.24	3.14	1.33	3045	16.26	3.12	1.29
428	0.94	3.13	1.35	3046	0.76	3.13	1.36
437	2.97	3.13	1.35	3047	23.68	3.09	1.32
439	3.56	3.13	1.35	3048	38.52	3.09	1.27
442	3.07	3.13	1.35	3049	10.23	3.11	1.35
444	4.61	3.11	1.37	3051	7.44	3.10	1.37
450	1.27	3.13	1.35	3056	7.92	3.11	1.35
452	2.73	3.12	1.37	3058	18.18	3.10	1.33
455	1.06	3.13	1.35	3061	7.42	3.10	1.37
457	0.62	3.13	1.36	3062	19.81	3.08	1.36
464	2.15	3.13	1.34	3068	11.19	3.10	1.36
466	6.12	3.14	1.31	3079	17.56	3.08	1.37
513	6.08	3.13	1.32	3080	6.71	3.11	1.36
534	5.01	3.13	1.34	3082	6.10	3.10	1.38
726	0.98	3.13	1.36	3091	5.96	3.10	1.38
1125	0.73	3.13	1.35	3151	3.74	3.13	1.34
1212	4.85	3.12	1.34	3159	3.75	3.12	1.35
1326	8.79	3.13	1.32	3177	0.40	3.12	1.36
1483	23.68	3.10	1.31	3180	0.45	3.12	1.37
1498	14.27	3.11	1.32	3182	3.81	3.12	1.35
2160	2.24	3.12	1.36	3192	0.77	3.13	1.36
2162	2.55	3.12	1.36	3229	2.80	3.11	1.37
2166	2.42	3.12	1.36	3479	4.62	3.13	1.33
2173	0.60	3.12	1.36	3487	0.97	3.12	1.37
2176	0.67	3.12	1.37	3506	2.20	3.13	1.35
2178	2.40	3.13	1.34	3514	1.58	3.13	1.36
2180	2.06	3.12	1.37	3546	4.37	3.12	1.35
2182	2.08	3.12	1.37	3550	4.68	3.12	1.35
2183	2.90	3.12	1.36	3558	0.77	3.13	1.35
2185	5.09	3.13	1.34	3646	3.04	3.12	1.35
2187	2.93	3.12	1.35	3701	5.87	3.14	1.32
2189	4.55	3.12	1.35	3710	13.93	3.15	1.27
2190	0.76	3.12	1.36	3714	11.53	3.15	1.27
2192	3.71	3.12	1.36	3716	11.03	3.12	1.32
2193	0.49	3.12	1.37	3765	37.63	3.16	1.15
2196	2.72	3.12	1.35	3766	32.27	3.14	1.19
2219	0.84	3.12	1.37	3965	7.95	3.14	1.30
2220	2.77	3.11	1.37	4891	0.97	3.12	1.37
2221	2.45	3.11	1.38	5156	0.40	3.12	1.37
2225	5.75	3.10	1.37	5330	1.14	3.13	1.35

**Table 3:** Significance of combined putative markers using forward regression, corrected for polygenic effects.

	markers	F-value	df e	var e	var a	h^2^	LogL	diff AIC
Round 0	---	---	4663	3.123	1.361	0.30	-5584	
								
Round 1	1483	13.25	4651	3.104	1.002	0.24	-5466	130
	3033	13.82						
	3048	9.32						
	3765	34.95						
								
Round 2	1483	15.94	4639	3.096	0.895	0.22	-5441	167
	3033	18.20						
	3048	2.35						
	3765	32.26						
	415	14.14						
	513	5.85						
	3031	4.58						
	3965	7.07						
								
Round 3	1483	11.09	4627	3.085	0.827	0.21	-5430	190
	3033	18.18						
	3048	2.78						
	3765	32.36						
	415	21.17						
	513	5.43						
	3031	4.75						
	3965	7.88						
	296	4.05						
	399	7.48						
	1326	7.85						
	2185	6.15						

**Table 4:** Genotypic effects of markers included in round 3 of the forward regression analysis (Standard errors of effects are given in italics).

genotype marker	11	12	21	22
296	0.000	0.050	0.134	0.307
	*0.000*	*0.118*	*0.122*	*0.121*
				
399	0.000	-0.316	-0.393	-0.551
	*0.000*	*0.118*	*0.123*	*0.122*
				
415	0.000	0.501	0.555	0.815
	*0.000*	*0.084*	*0.097*	*0.113*
				
513	0.000	-0.331	0.102	-0.008
	*0.000*	*0.094*	*0.099*	*0.146*
				
1326	0.000	0.223	0.164	0.449
	*0.000*	*0.095*	*0.097*	*0.099*
				
1483	0.000	-0.214	-0.442	-0.570
	*0.000*	*0.107*	*0.104*	*0.107*
				
2185	0.000	-0.198	-0.244	-0.472
	*0.000*	*0.078*	*0.092*	*0.115*
				
3031	0.000	0.212	0.363	0.616
	*0.000*	*0.105*	*0.119*	*0.226*
				
3033	0.000	0.400	0.574	0.980
	*0.000*	*0.098*	*0.118*	*0.133*
				
3048	0.000	0.234	0.152	0.299
	*0.000*	*0.100*	*0.116*	*0.141*
				
3765	0.000	0.490	0.556	1.004
	*0.000*	*0.091*	*0.094*	*0.102*
				
3965	0.000	-0.164	-0.286	-0.630
	*0.000*	*0.083*	*0.093*	*0.135*

**Figure 1:**
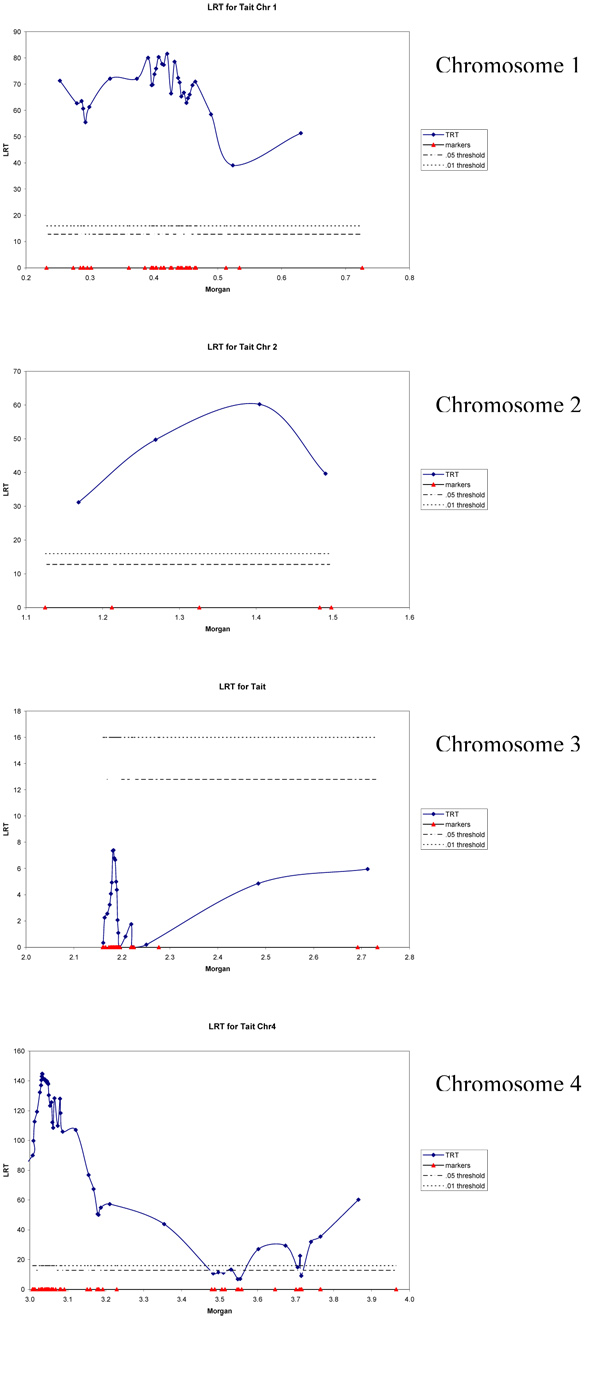
**Likelihood ratio profiles for chromosomes 1, 2, 3 and 4 with adjusted threshold**.

Subsequently LDLA was applied to these 114 markers and the profiles of the likelihood ratio test are shown in Figure 1 for chromosomes 1, 2, 3, and 4. Given these graphs and results from Table 3, two QTL are expected on chromosome 1, one QTL on chromosome 2, three or four QTL on chromosome 4 and none on chromosomes 3, 5, and 6.

Two very obvious candidates for further study were the regions between SNP 403 and SNP 466 on chromosome 1 and between SNP 3007 and SNP 3091 on chromosome 4. Both regions had a maximum log likelihood ratio greater than 80. QTLs with smaller effects are expected on chromosome 1 (to the left of SNP 232), on chromosome 2 (between SNP 1326 and 1483) and on chromosome 4 (between SNP 3646 and 3766 and around 3965).

The region on chromosome 3 around SNP 2185 did not show a peak in the LDLA-analysis. In this region sire 1 and 1034 were segregating (Table 1). Unlike the other regions, analysis on all sire families combined indicated that a QTL did not segregate in this region. Although the 2 sires segregated for 21 putative markers in a small region, the data did not support the presence of a QTL in this region. This is a clear example of a false positive putative QTL.

## Discussion

**Figure 2:**
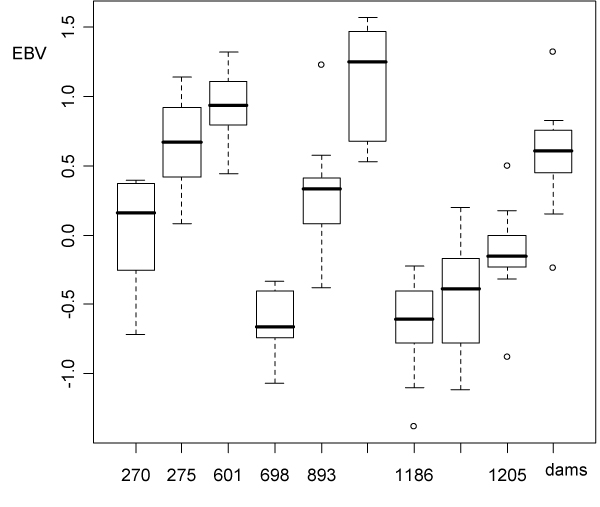
**Box-plot of estimated breeding values (EBV) of progeny of sire 389 by dam**.

The most critical part in selective genotyping strategies is to decide which animals should be included in high and low tails, as well as the number of tails that will be screened. In this data set there were marginal differences if the choice of animals was based on absolute value or on estimated breeding value. Under practical circumstances however the latter would be preferred. In this balanced data set the 10 best progeny (one per dam) were included in the high tail and the 10 worst (one per dam) were in the low tail. By choosing high/low within dam instead of across dams within sires, the chances of picking up false putative markers are reduced. Many more were found choosing across dams (data not shown). An illustration is the box-plot of estimated breeding values of progeny of sire 389 shown in Figure 2.

The data allowed for 45 high/low tails to be made because there were 45 sires with 100 progeny each. All tails were analyzed but only 21 sires showed segregation of at least one marker, nine of which were segregating for one marker only. A relevant question is whether the segregating sires could have been identified beforehand. It would decrease the work load for preparation and testing considerably. An analysis of higher moment statistics in the distribution of the phenotypes in the offspring might prove useful.

True positions of QTL were revealed after the workshop had taken place [7]. In Table 5 the estimated and true positions were compared. Eight of the 19 QTL (explaining 98% of the genetic variation) were found using our two-stage selective genotyping approach. About 54% of the genetic variance associated with these 19 QTL was covered by these eight QTL. Four additional QTL with smaller effects were also identified: S1 at 296 cM, S3/S4 at 513 cM, S21 at 3033 cM and S22 at 3048 cM. Additive QTL effects were not very well estimated, which might explain that some of the QTL with a smaller effect were not identified. The QTL at the beginning of chromosome 3 (SNP 2185), which was considered to be a false positive because it did not reach the significance level in the LDLA analysis, was in fact a QTL (M8) with a small effect.

**Table 5:** Simulated QTLs explaining more than 1% of the genetic variance and their true and estimated position and percentage of genetic (Va) and phenotypic (Vp) variance explained.

**rank**	**QTL**	**location**	**estimated**	**%Va**	**%Vp**
4	M1	200	296	0.12	0.03
12	M2	400	399	0.03	0.01
8	M3	772		0.04	0.01
7	M4	1274		0.05	0.01
13	M5	1300	1326	0.03	0.01
5	M6	1486	1483	0.05	0.02
6	M7	1749		0.05	0.02
15	S14	1935		0.02	0.01
17	S16	1978		0.01	0.00
11	M8	2149	2185	0.03	0.01
9	M9	2600		0.04	0.01
2	M10	3032	3033	0.13	0.04
10	M11	3369		0.03	0.01
3	M12	3761	3765	0.13	0.04
14	M13	3965	3965	0.02	0.01
16	M14	4052		0.01	0.00
18	S28	4684		0.01	0.00
19	S31	4770		0.01	0.00
1	M15	4935		0.18	0.05
					
		sum		0.98	0.29

The 2 stage approach reduced the number of genotypes from 28 million in the whole data set to 5.4 million in stage 1 plus 0.43 million in stage 2; a reduction of almost 80%. If SNP-genotyping allows for sufficient accurate estimation of allele frequency in pooled DNA, then only 540.000 genotypes have to be determined in the first stage, reducing the genotyping effort with another order of magnitude. The number of individuals to put into a pool depends on the accuracy of determining the allele frequency, which in turn depends on the method applied. With AFLP-markers the typical choice is to put 10 individuals in each pool [11].

## Conclusion

The two-stage genotyping strategy with selective genotyping detected regions with highly significant QTL useful for further fine-mapping. Large reduction of genotyping efforts saves costs which could be used for subsequent expression and functional analyses.

## Competing interests

The authors declare that they have no competing interests.

## Authors' contributions

HH and JB conceived the project. HH and SvdB analyzed the data. All took part in writing the paper.
